# The Impact of Early Brain-Dead Donor Detection in the Emergency Department on the Organ Donation Process in Iran

**DOI:** 10.3389/ti.2024.11903

**Published:** 2024-08-13

**Authors:** Arman Hasanzade, Seyed Mohammad Reza Nejatollahi, Mojtaba Mokhber Dezfouli, Mahdieh Hazrati, Soheil Sheikholeslami, Masoud Imani, Bardia Mohseni, Fariba Ghorbani

**Affiliations:** ^1^ Lung Transplantation Research Center, National Research Institute of Tuberculosis and Lung Diseases, Shahid Beheshti University of Medical Sciences, Tehran, Iran; ^2^ Hepato-Pancreato-Biliary and Transplant Surgery, Masih Daneshvari Hospital, Shahid Beheshti University of Medical Sciences, Tehran, Iran; ^3^ Organ Transplantation and Donation Research Center, National Research Institute of Tuberculosis and Lung Diseases, Shahid Beheshti University of Medical Sciences, Tehran, Iran; ^4^ Department of Biostatistics, School of Public Health, Iran University of Medical Sciences, Tehran, Iran; ^5^ Tracheal Diseases Research Center, National Research Institute of Tuberculosis and Lung Diseases, Shahid Beheshti University of Medical Sciences, Tehran, Alborz, Iran

**Keywords:** organ donation, emergency department, donation policy, donor identification, brain dead donor, donor detection, conversion rate, hospital characteristics

## Abstract

We aimed to assess the impact of hospital characteristics on the outcomes of detected possible brain-dead donors, in our organ procurement network in Iran. Data was collected through twice-daily calls with 57 hospitals’ intensive care units and emergency departments over 1 year. The donation team got involved when there was suspicion of brain death before the hospital officially declared it. The data was categorized by hospital size, presence of neurosurgery/trauma departments, ownership, and referral site. Out of 813 possible donors, 315 were declared brain dead, and 203 were eligible for donation. After conducting family interviews (consent rate: 62.2%), 102 eligible donors became actual donors (conversion rate: 50.2%). While hospital ownership and the presence of trauma/neurosurgery care did not affect donation, early referral from the emergency department had a positive effect. Therefore, we strongly recommend prioritizing possible donor identification in emergency rooms and involving the organ donation team as early as possible. The use of twice-daily calls for donor identification likely contributed to the consistency in donation rates across hospitals, as this approach involves the donation team earlier and mitigates the impact of hospital characteristics. Early detection of possible donors from the emergency department is crucial in improving donation rates.

## Graphical Abstract

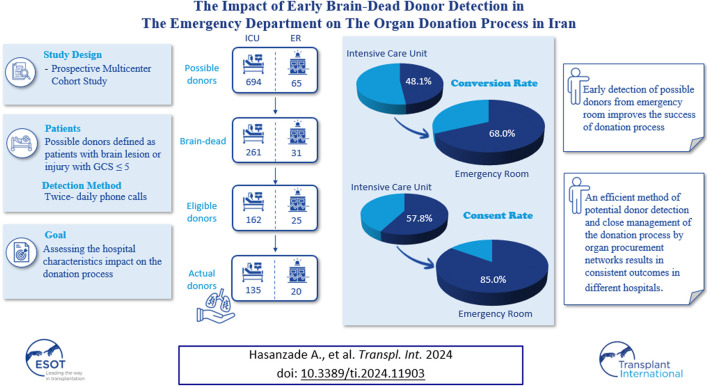

## Introduction

Organ transplantation is the most effective treatment in end-stage organ diseases [1]. Despite numerous efforts to increase the global donation rates, the gap between the demand and supply of organs is increasing due to the rising incidence of organ failure diseases [2]. The most significant limitation of donation is limited donor pool [3, 4]. While, organs can be recovered from living donors and from donors after circulatory death, still significant proportion of donation are dependent on brain dead donors (BDDs) [1, 2]. In the United States in 2021, there were 30,874 BDDs, which is much higher than the 6,539 living donors [5].

The process of donation from BDD is complex. The first step is identification of the possible donor [1]. A possible donor is a patient with brain lesion or injury, having a Glasgow Score (GCS) less than 5 or 8, according to the policy of jurisdiction [1, 6, 7].

The condition of possible donors may either improve or deteriorate. If in any possible donor, deep coma (GCS = 3) occurs, evaluation of brain death should be considered. A potential donor is a patient, whose condition is suspected to meet the criteria for brain death [7]. The evaluation of brain death involves serial examinations for coma and brainstem reflexes over at least 6 h, as well as ancillary tests [8, 9]. According to the American Academy of Neurology, brain death is an irreversible loss of brainstem and brain functions, confirmed by permanent coma, apnea, and brain stem reflexes absence [9].

However, not all brain-dead potential donors meet the criteria for eligible donor. An eligible donor is a legally declared brain-dead patient who is medically suitable for donation and has no contraindication of donation, with the criteria defined by the related jurisdiction [7]. For example, according to the Organ Procurement and Transplantation Network Policy, these criteria consist of age ≤ 75, weight > 5 kg, and a body mass index ≤ 50 kg/m^2^, without any exclusion criteria such as demonstrating any neoplastic or infectious disease risk for the recipient [5]. An Actual donor is an eligible consented donor from whom at least one organ was recovered for donation, or at least a surgical incision was made with the purpose of organ recovery for transplantation [7, 10].

In addition, managing potential donors and family interview are other crucial steps in the organ donation process [8, 10]. Therefore, organ donation is a multi-step process, and loss of brain-dead donors and organs can occur at any stage. Failure to identify potential donors, donor circulatory death, ineffective management and family refusal are the main reasons of failure [3, 8, 10].

In Iran, we have 24 Organ Procurement Units and more than 60 BDD detection units. In 2022, the donation rate was 12.2 PMP and 2,234 organ transplantations were performed from deceased donors, mostly from donation after brain death rather than circulatory death. For instance, out of 1,016 actual deceased donors in 2022, only 3 were from circulatory death [11]. Hence, donation after brain death holds significant importance. Living donation is also common in Iran, and some of these living donations are in exchange for compensation. While this issue is not prohibited by law, deceased donation in exchange for money is illegal, with strict surveillance. Over the past decade, efforts have been made to decrease living donation and promote donation after brain death. The number of living donors decreased from 1,540 in 2013 to 1,276 in 2022, while deceased donors increased from 670 to 1,016 [11, 12]. Therefore, it is important to investigate the process of donation after brain death and strive for improvement.

Hospitals policies and staff play an important role in this process, and donation rates vary among different hospitals. Some hospital characteristics are associated with higher donation rate such as larger size, being trauma center, having more intensive care unit (ICU) beds, having neurology and neurosurgery department, being an academic hospital, and being located in an urban area [10, 13–15]. Therefore, in this study, we aimed to evaluate our method of early identification of possible donors and the hospitals characteristics that may affect donation process in our Organ Procurement Network (OPN).

## Materials and Methods

### Our OPN Protocol

In Iran, the majority of deceased donations come from brain-dead donors, and the process of donation from BDDs begins with the detection of possible donors. The identification of possible donors within this OPN involves five well-trained and experienced coordinators initiating telephone calls to the ICUs and Emergency Departments (EDs) of the 57 affiliated urban hospitals, conducted twice daily. During these calls, we inquire with the head nurse about any patients with a GCS≤ 5 in their ward. Their responses rely on examinations by the attending physicians, predominantly intensivists, neurologists, internists, or neurosurgeons. Notably, all ICU and ED nurses in these hospitals are trained in the field of brain death and organ donation, having successfully passed a training course examination. Also, their reports undergo random checks through unannounced visits by our supervisors.

Beyond the ED and ICU, hospitals are obligated to report if any possible donor is identified in other hospital wards. In such cases, that specific ward is included in our twice-daily calls to monitor the possible donor. Notably, promptly transferring possible donors to the ICU from various wards and the ED is mandatory to ensure supervision by both intensivists and attending physicians.

Patients reported with GCS ≤ 5 are enrolled in our database. Subsequent calls track these possible donors until one of three events: improvement in the patient’s condition and consciousness, circulatory death, or a decrease in the consciousness.

For patients with GCS = 3, a coordination team is sent, as there are no donation professionals at hospitals. A comprehensive neurologic examination, including GCS, brain stem reflexes, and the apnea test, is conducted. These examinations are carried out separately by the neurologist or intensivist (attending physician) of the center and the coordinator. To ensure the irreversibility of the loss of brain stem reflexes, the assessment should be carried out for at least 6 h according to this jurisdiction’s law. Brain death is declared only when both attending physician and donation team agree on the diagnosis. Following the brain death, potential brain-dead donors are assessed for donation eligibility criteria. For those eligible donors, the viability of organs, is examined for donation.

Physicians and coordinators jointly attend the family interview when delivering the bad news regarding brain death. However, neither discusses donation. Following this, a period is given to the family to grieve and believe the death. Throughout this time, coordinators engage with the family, fostering a supportive relationship. If the coordinator senses that the family has accepted the death, they cautiously mention the donation. It’s notable that the family will approach only if at least one organ of the eligible donor is viable for donation. Throughout this process, donor management takes place in the ICU, supervised by both the hospital intensivist and this OPN.

Eligible donors, whose families have consented for donation, are transferred to the OPN. Following the jurisdiction’s protocol outlined by the Ministry of Health, four physicians who are affiliated to Ministry of Health (an internist, a neurosurgeon, a neurologist, and an intensivist) are randomly assigned to the eligible donor to confirm brain death, once more. Additionally, ancillary tests such as two EEG by the interval of 6 h and according to the clinical features, transcranial doppler ultrasound, or four-vessel computed tomography angiography maybe performed. Organ allocation occurs after brain death is confirmed by these physicians, and the allocation process is overseen by the Ministry of Health.

#### Study Design and Setting

This prospective cohort study was conducted at Masih Daneshvari Organ Procurement Network based in Iran, aimed to evaluate hospital characteristics influencing the donation process. The study received approval from the ethics committee of the National Research Institute of Tuberculosis and Lung Diseases with reference number IR.SBMU.NRITLD.REC.1402.058. Data pertaining to all possible donors registered in the detection database were extracted from January to December 2022. These data encompass hospital and possible donor characteristics, along with the outcome of the donation process for each potential donor.

Independent variables:

1. Hospital characteristics:
• 29 Private hospitals vs. 27 public hospitals.
• The number of beds in ICU, neurosurgery and neurology ward, with the range of 14–200.
• 20 hospitals providing both trauma and neurosurgery care are defined as type I, while 37 hospitals with no trauma and neurosurgery care are considered type II hospitals. It’s notable that there is no hospital connected to our OPN that only has one of the mentioned departments.
• The referral site including ICU or Emergency Department. While possible donors are detected in other hospital wards some instances, due to the variety of these wards and lower number of detected potential donors, we only compared the donation process between ICU and ED.
2. Possible donor characteristics including age, gender, cause of loss of consciousness (LOC), final outcome and follow-up duration.

The follow-up duration is the time from the detection of possible donors to the occurrence of one of the three outcomes (improvement, circulatory death, or the first diagnosis of brain death). Therefore, the period of monitoring the irreversibility of brain death for 6 h and the donation process, from evaluating eligibility criteria to organ recovery, is not included in this term.

Outcomes:

• The conversion rate was calculated by dividing the number of actual donors by the number of eligible donors.
• The actual Donor to Brain Dead Ratio (AD/BD) is a measure that indicates the proportion of brain-dead potential donors from whom organ donation occurred.
• The Eligible Death Ratio (EDR) was defined as the ratio of the number of eligible donors to the total number of possible donors who have died, whether due to circulatory death or brain death.
• The Organ Loss Ratio (OLR) is a measure that reflects the proportion of eligible donors from whom no suitable organs could be donated.
• The Consent rate considered as the proportion of obtained consents from families interviewed.

### Statical Analysis

The data collected in Google Sheets were exported to SPSS version 25 for this study. Descriptive evaluations were presented as mean ± standard deviation (SD) for quantitative variables and frequency (percentage) for categorical variables. The effects of hospital characteristics, including private vs. public and type I vs. type II hospitals, as well as referral of possible donors from ED vs. ICU, on the five mentioned outcomes were analyzed using Chi-square test and reported using Risk Ratio (RR), 95% Confidence Interval (CI), and P-value. To assess the impact of hospital size and follow-up duration on the binomial outcomes, Logistic Regression was employed, and the results were described by Odds Ratio (OR), CI, and P-value.

## Results

### Study Population

Between January 1st, and December 31st, 2022, 813 possible donors were enrolled. The baseline characteristics of these possible donors, including age, gender, patient’s outcome, follow-up duration, the detection site, hospital characteristics, and donation outcomes, are fully detailed in Table 1. Furthermore, the data related to the donation process, categorized by hospital characteristics, is mentioned in Table 2.

**TABLE 1:** Baseline Characteristics and Outcomes of 813 Possible Donors.

		Mean ± SD
Age (years)		42.3 ± 18.8
Follow-Up Duration (days)		4.53 ± 8.6
		N (%)
Male (%)		539 (66.3)
Cause of LOC	Trauma	153 (18.8)
	Poisoning	134 (16.5)
	Cerebrovascular Accident	244 (30.0)
	Brain Tumor	71 (8.7)
	Hypoxemia	131 (16.1)
	Other	79 (9.7)
Hospital Characteristics	Type I	766 (94.6)
	Type II	44 (5.4)
	Public	714 (88.1)
	Private	96 (11.9)
Detection Site	ICU	694 (85.6)
	ED	65 (8.1)
	Other wards	51 (6.3)
Patient Outcome^b^	Improvement	243 (29.9)
	Circulatory Death	247 (30.4)
	Brain Death	315 (38.7)
Donation Details of Potential Brain-Dead Donors^a^	Eligible Donors	203 (64.4)^†^
	No Viable Organ	35 (11.1)^†^
	Consent to Donate	102 (32.4)^†^
	Actual Donors	102 (32.4)^†^

^a^ : The reported percentages pertain to the entire pool of potential brain-dead donors (315). The key studied ratios providing a better understanding of the donation process are as follows: Conversion Rate: 50.2%, Actual Donor/Brain Dead: 32.4%, Eligible Death Ratio: 36.1%, Organ Loss Ratio: 17.2%, Consent Rate: 62.2%.

^b^ : Unfortunately, data on the follow-up of 8 possible donors were not recorded.

**TABLE 2:** Outcomes of Possible Donors Based on Hospital Characteristics and Detection Site.

		Hospital types		Public vs. Private		Detection Location^a^	
		Type I	Type II	Public	Private	ICU	ER
N (%)		766 (94.6)	44 (5.4)	714 (88.1)	96 (11.9)	694 (85.6)	65 (8.1)
Possible Donors	Improvement (%)	234 (30.5)	9 (20.5)	222 (31.1)	21 (21.9)	220 (31.7)	13 (20.0)
	Circulatory Death (%)	230 (30.0)	17 (38.6)	220 (30.8)	27 (28.1)	208 (30.0)	21 (32.3)
	Brain Death (%)	297 (38.8)	18 (40.9)	268 (37.5)	47 (49.0)	261 (37.6)	31 (47.7)
Brain Dead Potential Donors	Eligible (%)^b^	192 (64.6)	11 (61.1)	175 (65.3)	28 (59.6)	162 (62.1)	25 (80.6)
	Ineligible (%)^b^	105 (35.4)	7 (38.9)	93 (34.7)	19 (40.4)	99 (37.9)	6 (19.4)
Eligible Donors	No Viable Organ (%)^c^	33 (17.2)	2 (18.2)	31 (17.7)	4 (14.3)	25 (15.4)	3 (12.0)
	Refuse to Donate (%)^c^	58 (30.2)	4 (36.4)	54 (30.9)	8 (28.6)	57 (35.2)	3 (12.0)
	Actual Donor (%)^c^	97 (50.5)	5 (45.5)	86 (49.1)	16 (57.1)	78 (48.1)	17 (68.0)

^a^ : 51 (6.3%) from other hospital wards.

^b^ : Percentages are reported among brain-dead potential donors, not overall possible donors in each category

^c^ : Percentages are reported among eligible donors, not overall possible donors in each category. Unfortunately, data on the follow-up of 8 possible donors were not recorded. Seven of them were from Type I Public hospitals, and 1 was from Type I private hospital. Additionally, 7 of them were detected in the ICU, and 1 in the ED.

### Analysis of Donation Process Outcomes

#### Type I vs. Type II Hospitals

**FIGURE 1:**
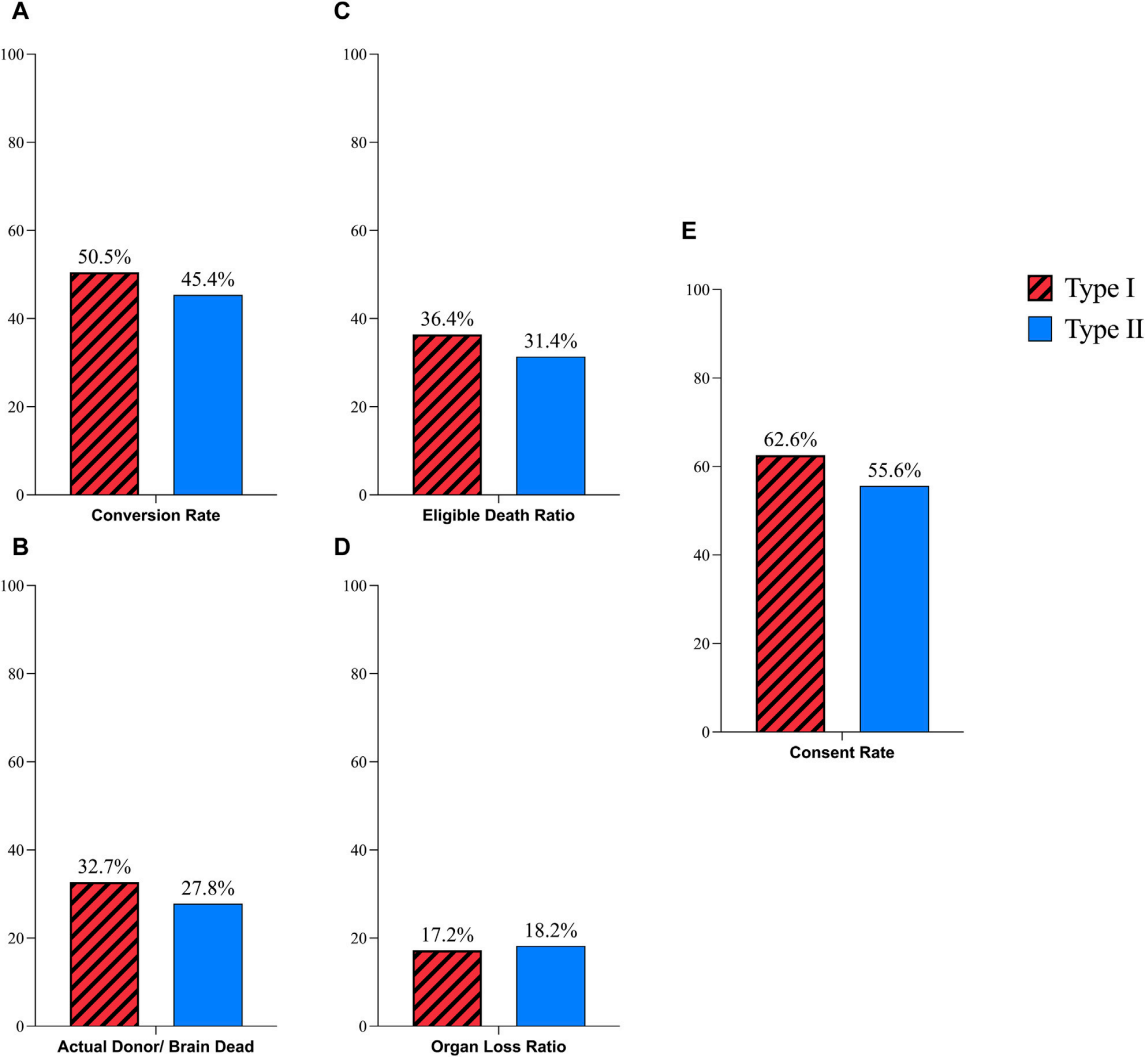
Comparison of donation process in Type I and Type II hospitals; **(A)** Coversion Rate; **(B)** Actual Donor/Brain Dead Ratio; **(C)** Eligible Death Ratio; **(D)** Organ Loss Ratio; **(E)** Consent Rate.

The conversion rate in type I hospitals was 50.5% which was not statistically different from type II hospitals (45.5%) (P-Value: 0.74, RR: 1.11, CI: 0.57–2.15). The AD/BD ratio was 32.7% in type I hospitals and 27.8% in type II hospitals, with no significant difference (P-Value: 0.66, RR: 1.18, CI: 0.55–2.52). The EDR was 36.4% in type I and 31.4% in type II hospitals, with no significant difference observed (P-Value: 0.55, RR: 1.15, CI: 0.7–1.91). The OLR was not significantly different between two types, Type I 17.2% and Type II 18.2% (P-Value: 0.93, RR: 0.94, CI: 0.26–3.43). In type I hospitals, from 155 approached families 97 (62.6%) families consented to donation. In type II hospitals, the consent rate was 55.6% from 9 interviewed families. However, statistical analysis indicated no significant difference (P-Value: 0.67, RR: 1.12, CI:0.62–2.04), (Figures 1, 4A).

#### Public vs. Private Hospitals

**FIGURE 2:**
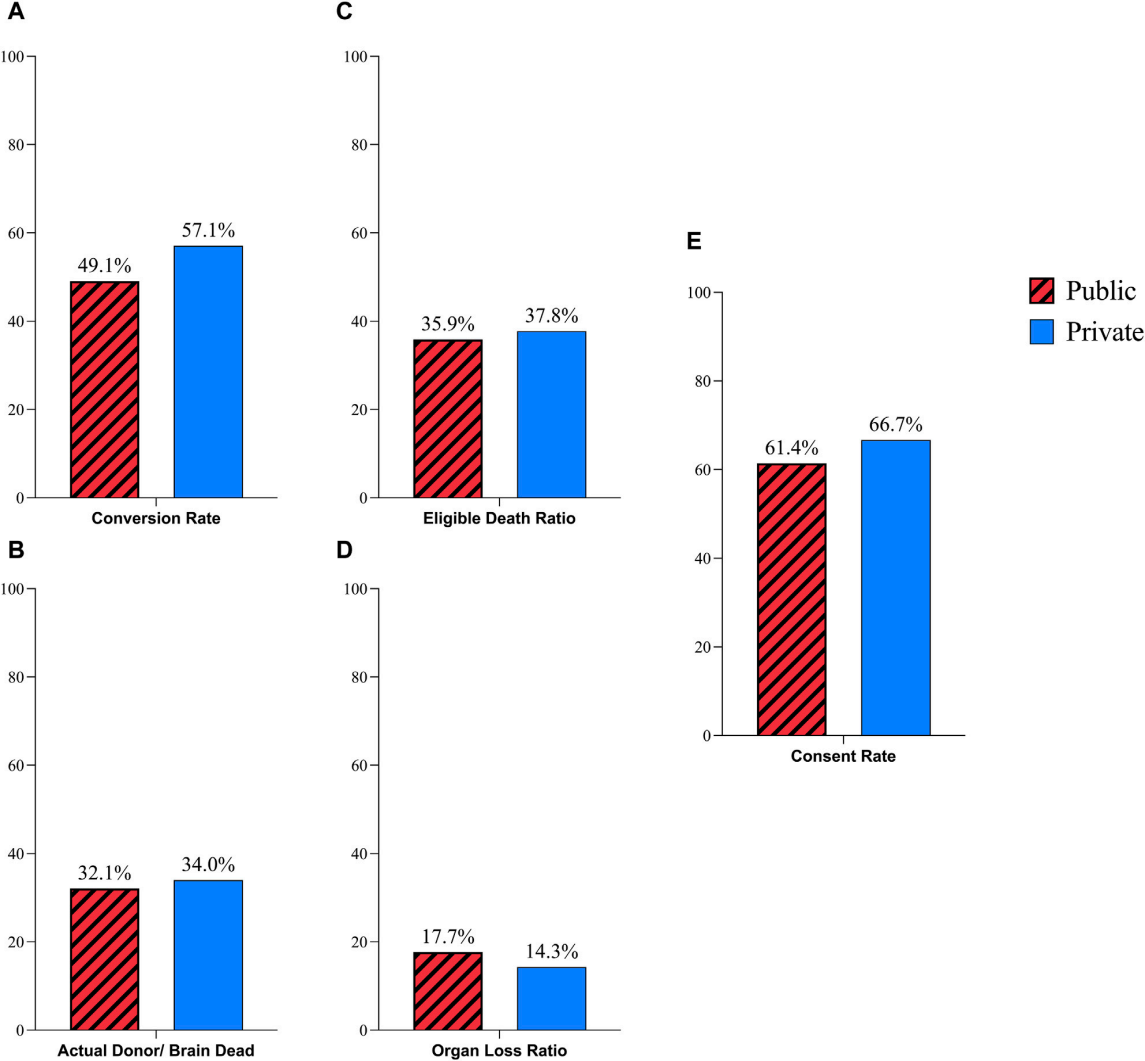
Comparison of donation process in public and private hospitals; **(A)** Coversion Rate; **(B)** Actual Donor/Brain Dead Ratio; **(C)** Eligible Death Ratio; **(D)** Organ Loss Ratio; **(E)** Consent Rate.

Public hospitals conversion rate was 49.1% which in comparison with 57.1% rate of private hospitals was not significantly different (P-Value: 0.43, RR: 0.86, CI: 0.6–1.22). The difference between the AD/BD ratio of public hospitals (32.1%) and private hospitals (34%) was not meaningful (P-Value: 0.79, RR: 0.94, CI: 0.61–1.45). The OLR was 17.7% in public hospitals while this ratio was 14.3% in private hospitals which did not show considerable difference. (P-Value: 0.65, RR: 1.24, CI: 0.47–3.24). There was no significant difference in the EDR between public hospitals (35.9%) and private hospitals (37.8%) (P-Value: 0.74, RR: 0.94, CI: 0.69–1.29). The difference of public hospitals consent rate (61.4%) and private hospitals (66.7%) was not statically significant as well (P-Value: 0.62, RR: 0.92, CI: 0.67–1.25), (Figures 2, 4B).

#### ICU vs. ED

**FIGURE 3:**
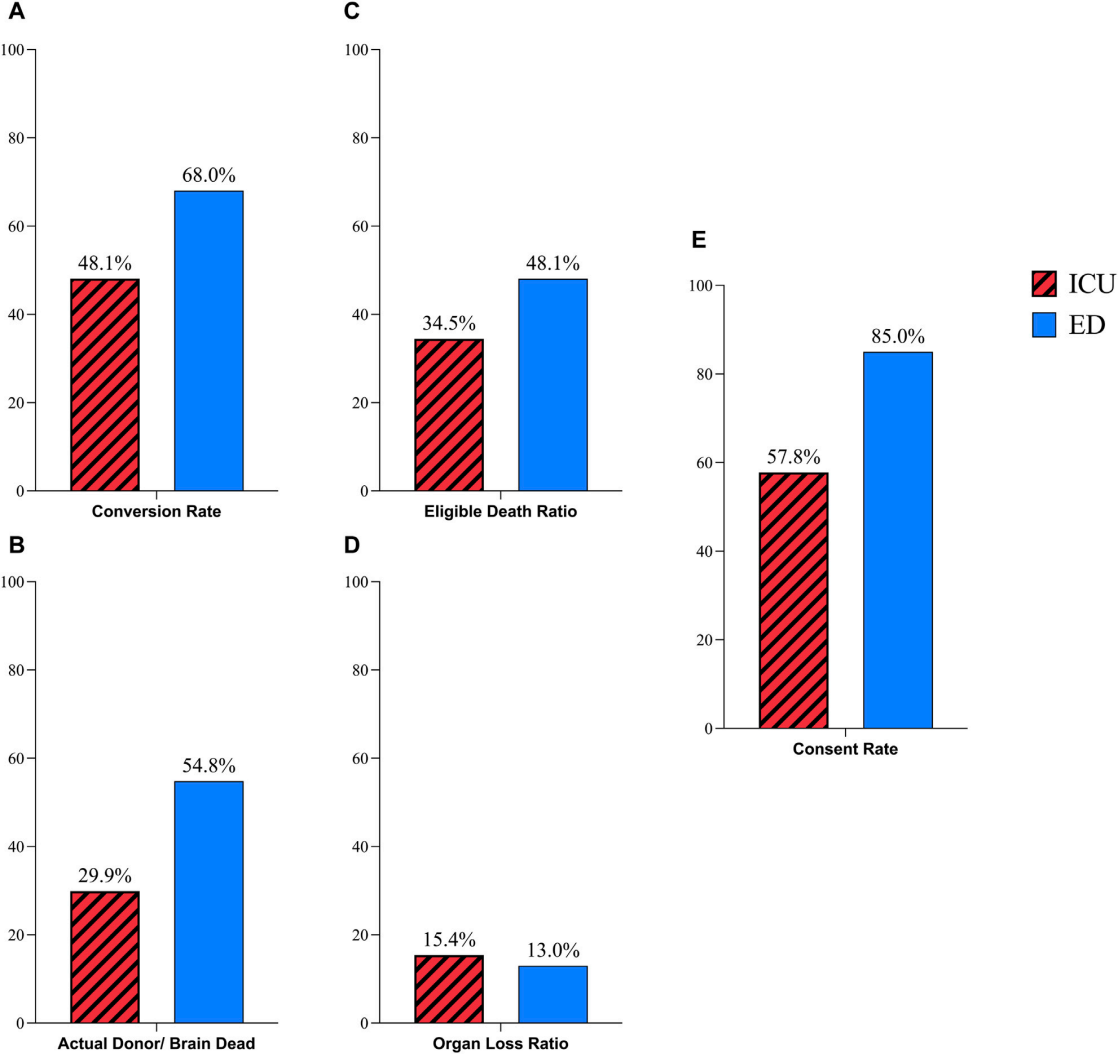
Comparison of donation process in ICU and ED referred possible donors; **(A)** Coversion Rate; **(B)** Actual Donor/Brain Dead Ratio; **(C)** Eligible Death Ratio; **(D)** Organ Loss Ratio; **(E)** Consent Rate.

**FIGURE 4:**
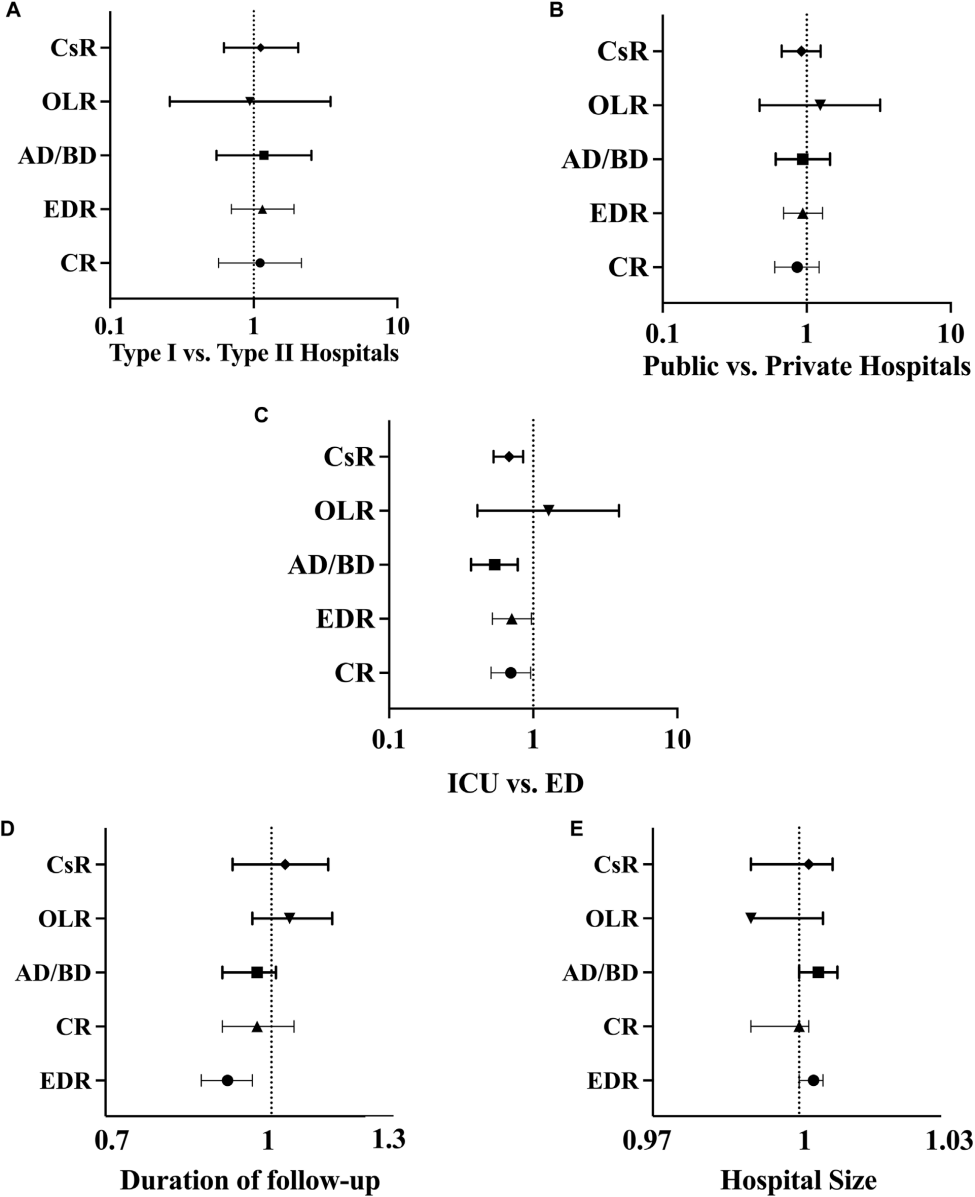
Forest plot for univariable analysis: **(A)** Comparison of donation process in type I and type II hospitals; **(B)** Comparison of donation process in public and private hospitals; **(C)** Comparison of donation process in ICU and ED referred possible donors; **(D)** Analysis of the impact of follow-up duration on different outcomes; **(E)** Analysis of the impact of hospital size on different outcomes. Abbreviations: CR, conversion rate; AD/BD, actual donor/brain dead; EDR, eligible death ratio; OLR, organ loss ratio; CsR, consent rate; ICU, intensive care unit; ED, emergency department.

The conversion rate in patients referred from EDs was 68%, which showed a nearly significant difference compared to the conversion rate of 48.1% in ICU-referred patients (P-Value: 0.065, RR: 0.7, CI: 0.51–0.96). The AD/BD ratio in EDs was 54.8%, which was significantly higher than the ratio of 29.9% in ICUs (P-Value: 0.005, RR: 0.54, CI: 0.37–0.78). Another ratio that demonstrated a statistically difference between ICU and ED was the EDR which was 48.1% in EDs and 34.5% in ICUs (P-Value: 0.05, RR: 0.71, CI: 0.52–0.97). The consent rate also showed a meaningful difference, with a rate of 85% in EDs and 57.8% in ICUs (P-Value: 0.02, RR: 0.68, CI: 0.53–0.85). The only ratio that showed no significant changes between these two referral sites was the OLR that was 13% in EDs, and 15.4% in ICUs (P-Value: 0.65, RR: 1.28, CI: 0.41–3.94), (Figures 3, 4C).

#### Follow-Up Duration

The follow-up duration, showed no influence on the evaluated ratios except for the EDR (P-Value<0.01, OR: 0.91, CI: 0.86–0.96). The evaluation indicated that the chance of eligible death decreases with each day of increase in follow-up duration. Regarding the other ratios, including conversion rate (P-Value: 0.51, OR: 0.97, CI: 0.9–1.05), OLR (P-Value: 0.29, OR: 1.04, CI: 0.96–1.14), consent rate (P-Value: 0.63, OR: 1.03, CI: 0.92–1.13), and the AD/BD ratio (P-Value: 0.39, OR: 0.97, CI: 0.9–1.008), there were no significant associations observed with follow-up duration (Figure 4D).

#### Hospital Size

The results showed no significant relationship between hospital size and conversion rate (P-Value: 0.49, OR: 1.002, CI: 0.99–1.00), OLR (P-Value: 0.70, OR: 0.99, CI: 0.99–1.005), and consent rate (P-Value: 0.49, OR: 1.002, CI: 0.99–1.007). However, there were near significant changes for the EDR (P-Value: 0.07, OR: 1.003, CI: 1.00–1.005) and the AD/BD ratio (P-Value: 0.06, OR: 1.004, CI: 1.00–1.008). The statistics indicated that with an increasing number of hospital beds, these ratios increased. Although these changes were not significant, there was a trend suggesting a potential impact of hospital size (Figure 4E).

## Discussion

Our findings indicate that, although being a public hospital and a type I hospital are associated with a higher number of potential donors, they do not have an impact on the success of any stage in the donation process in this OPN. Accordingly, the likelihood of a potential donor progressing to become an actual donor is equal regardless of these characteristics. Furthermore, our study reveals that referring possible donors from ED, improves the donation process. This finding highlights the importance of early involvement of the OPN. We also did not observe any relationship between hospital size and the success of donation. This is promising, as it suggests that smaller hospitals, despite having fewer potential donors and possibly less familiarity with the donation process, still offer an equal chance of donation for potential donors.

Studies suggested identification of potential donors from ED instead of ICU leads to expansion of donor pool [16, 17]. A previous study concluded that identifying potential donors in the ED not only increases the number of potential and actual donors but also leads to a higher ratio of organs donated per donor (3.79) compared to ICU (3.16) [18]. Another study, found a lower refusal rate among potential donors referred from the ED (33.5% vs. 42.7%) [19]. A subsequent systematic review confirmed that the chances of becoming actual donors are higher among patients referred from the ED [20]. Consistent with previous studies, our findings, demonstrate not only a significantly higher consent rate in the ED but also a higher rate of eligible deaths, a higher ratio of actual donors to brain-dead patients and a higher conversion rate. We speculate that the higher consent rate in the ED could be attributed to earlier efforts to establish a better relationship with the families. Importantly, we found no difference in the organ loss ratio. This lack of difference is reasonable since patients referred from the ED would be transferred to the ICU, and the management would continue in a similar manner. In conclusion, we strongly recommend considering organ donation and referral to organ procurement organizations in emergency departments.

The majority of possible donors require neurosurgical and trauma care. Therefore, it was expected that hospitals with neurosurgery/trauma departments would have a higher number of potential donors. This assumption has been supported by various studies, including the present paper. Neurosurgery department have been associated with an expansion of the pool of possible donors [21] and trauma center hospitals have shown higher numbers of both eligible and actual donors [13, 14]. Hence, it is crucial not to overlook other hospitals. It is essential to improve the donation process in all hospitals, irrespective of the presence of trauma/neurosurgery care. Furthermore, we expected that with efficient donation policies, there should be an equal chance of donation for potential donors in different hospitals. Contrary to our expectations, previous studies have shown a higher conversion rate in trauma centers [22–24] as well as higher consent rates [24]. The presence of trauma surgeons has also been found to increase the conversion rate [25]. Unexpectedly, Rios Diaz et al. found a higher conversion rate in non-trauma centers [26]. Since none of the hospitals evaluated in our study had solely a trauma or neurosurgery department, we were compelled to assess the effect of the existence of both departments together. While we confirmed a higher number of possible, potential, eligible, and actual donors in type I hospitals, our findings demonstrate no significant difference in the success of the donation process. We speculate that our methodology, which involved detecting possible donors through twice-daily calls, closely following the condition of possible donors, and handling further steps with the assistance of our coordinators once the patient was declared brain dead, may have contributed to similar success rates in hospitals regardless of the presence of neurosurgery/trauma care. However, this is only a suggestion and should be further assessed in future studies.

In Iran, the lower cost of care in public hospitals results in more admission. Subsequently, it is expected that public hospitals would have a higher number of potential donors. While there are few studies comparing organ donation between public and private hospitals in other countries, an assessment of kidney donation rates in South Africa showed a higher rate of donation in private hospitals [27], while the consent rate was higher in public hospitals [28]. Another study conducted in the United States found no difference in the conversion rate based on hospital ownership [26]. We argue that even with a smaller number of potential donors in private hospitals, the donation process should be of the same quality. Our evaluation found no difference in the rates of donation, consent, eligible deaths, or organ loss. We hypothesize that our method of identification and management of possible donors may have contributed to this promising finding.

Larger hospitals often have a higher rate of admission and increased availability of resources and equipment. It is expected that these advantages would lead to a higher number of potential donors, which was supported by our study and Roggenkamp et al. [13]. Lynch et al. obtained similar results for the number of eligible deaths [29]. However, a higher number of potential donors does not necessarily translate to a better success of donation. Our analysis revealed no relationship between the number of ICU, neurosurgery and neurology ward beds with the consent rate, conversion rate, or the ratio of eligible deaths. Similar conclusions were drawn in Webster et al.'s evaluation of the effects of pediatric intensive care unit size on donation [14]. Contrary to our desirable findings, some studies found higher conversion rates [24, 26] and higher consent rates [24] in smaller hospitals. Conversely, Domingo’s et al. found higher conversion rates in larger hospitals [30]. Again, we found the alternation of donation success with hospital characteristics, an undesirable outcome. We suggest improving policies to increase organ donation rates regardless of hospital characteristics.

As mentioned, contrary to our findings, numerous previous studies have reported the influence of hospital characteristics on the donation process. While the underlying reason for this favorable outcome requires further investigation, we assume that our method of twice-daily calls for the detection of possible donors and further follow-up resulted in the homogenization of the donation process in different hospitals. Our method differs from the donation models utilized in countries with high donation rates such as Spain [31], the United Kingdom [32], and Croatia [33, 34]. In these models, transplant coordinators or specialist nurses operate at the hospital level to identify possible donors, educate hospital staff, interview potential donor families, and manage other steps of the donation process. These methods are highly dependent on the coordination team within each hospital. Conversely, in the model utilized in our OPN, all these activities are mainly performed by the OPN with the cooperation of the medical team at hospitals (physicians and nurses). While comparing these models is not the purpose of this paper, our model appears to be efficient with lower costs than the mentioned methods, particularly in possible donor identification. However, further investigation is necessary to better understand these differences.

Additionally, when evaluating our OPN data for 2022 compared to 2009, we observed an increase in the utilization rate (utilized donor/actual donor) from 85% to 94% [35] Furthermore, although unpublished, over the 19-year activity period of this OPN, we have noted a rise in the overall consent rate from 30% to 85%, attributed to enhancements in our donation methods, including greater involvement of donation coordinators. While promising, further investigation is necessary to evaluate our model, particularly the efficacy of the twice-daily calls method in detecting potential donors.

Lastly, it’s noteworthy that various etiologies can lead to brain death, but not all cases are considered eligible. Death resulting from trauma has generally been associated with a higher rate of donation. While our investigation showed a near significant difference only in the eligible death ratio and not in the conversion rate, several studies have reported otherwise. In two previous studies, trauma-related cases having the highest conversion rates compared to other etiologies [22, 30].

Our study had some limitations. Donation is a complex process. Although we attempted to evaluate some of the hospital-related factors, many factors did not consider including cultural and religious factors. In our study, we only assessed donation process, using our twice-daily calls methodology for possible donor identification. Therefore, our assumption of superiority of this method is only a hypothesis, and future studies needed to compare this method with other models. For example, the presence of key donation professionals in the hospital, which we do not have.

In conclusion, we strongly recommend an early approach of identifying potential organ donors in emergency departments, which has the potential to significantly improve referrals to organ procurement organizations. Additionally, we emphasize the importance of implementing effective policies for the possible donor identification, closely monitoring their condition, and providing supervision over their management. By doing so, we believe that every potential donor in different hospitals should have an equal chance of donation. In total, while many studies have mentioned the early involvement of the donation team, we believe that our approach, leading to the early engagement of the organ donation team, has been instrumental in ensuring a consistent quality of the donation process across hospitals connected to our OPN. Furthermore, we conclude that the referral of possible donors from ED significantly enhances the donation. Therefore, it is essential for hospitals to consider training ED nurses and physicians to improve the identification of possible donors.

## Data Availability

The original contributions presented in the study are included in the article/supplementary material, further inquiries can be directed to the corresponding author.

## Abbreviations

OPN, Organ Procurement Network; ICU, Intensive Care Unit; ED, Emergency Department; GCS, Glasgow Score; LOC, Loss Of Consciousness; CVA, Cerebrovascular Accident; BDD, Brain Dead Donor; CR, Conversion Rate; EDR, Eligible Death Ratio; OLR, Organ Loss Ratio; CsR, Consent Rate; RR, Risk Ratio; OR, Odds Ratio; CI, Confidence Interval.
